# The Significance of Off-Season Tailor-Made Baseline Measurements in the Assessment of Post-Concussion in University Athletes

**DOI:** 10.3390/brainsci14070698

**Published:** 2024-07-12

**Authors:** Kyosuke Goto, Yutaka Shigemori, Yoshitaka Tanabe, Shunya Otsubo, Nana Otsuka, Koki Terada, Rino Tsurusaki, Keita Yamaguchi, Kentaro Masuda, Hiroshi Fukushima, Muneyuki Tachihara, Hironobu Shimozono, Jun Murakami

**Affiliations:** 1 Graduate School of Sports and Health Science, Fukuoka University, Fukuoka 814-0180, Japan; goto_411@yahoo.co.jp (K.G.); tanabe.yoshi0813@icloud.com (Y.T.); n.otsuka0419@fukuoka-u.ac.jp (N.O.); kokitrd21@gmail.com (K.T.); rino@ip.kyusan-u.ac.jp (R.T.); keita_1121@icloud.com (K.Y.); jinsg-627tbs@outlook.jp (K.M.); ffha29@gmail.com (H.F.); mune.tachihara@gmail.com (M.T.); 2 Department of Rehabilitation, Fukuoka University Hospital, Fukuoka 814-0180, Japan; 3 Faculty of Sports and Health Science, Fukuoka University, Fukuoka 814-0180, Japan; cmo@adm.fukuoka-u.ac.jp (H.S.); jun-m@adm.fukuoka-u.ac.jp (J.M.); 4 Center for Education and Innovation, Sojo University, Kumamoto 860-0082, Japan; otsubo@m.sojo-u.ac.jp

**Keywords:** sports-related concussion (SRC), sports concussion assessment tool, tailor-made baseline

## Abstract

This study aimed to investigate the significance of baseline measurements for amateur team athletes playing contact and collision team sports with a specific focus on the Sports Concussion Assessment Tool (SCAT) to improve concussion management. Symptoms of sports-related concussions (SRCs) can be diverse and long-lasting and include cognitive impairment, sleep disturbances, and vestibular dysfunction. Therefore, comprehensive baseline data are essential to preventing recurrent concussions and secondary injuries. This study was conducted during the 2023 off-season and evaluated the baseline condition of 65 male university rugby players using the SCAT5, which includes self-reported symptoms, and the modified Balance Error Scoring System (mBESS). The athletes were assessed for the presence or absence of SRC, and the mean values were compared using the Mann–Whitney U test. Among the participants, 35.38% (23/65) reported symptoms, with an average of 1.5 ± 2.8 symptoms per player and an average symptom score of 2.66 ± 5.93. In the mBESS, no errors were observed in the tandem stance test; however, 72.31% (47/65) made errors in the single-leg stance test on the non-dominant foot, with an average of 1.7 ± 1.5 errors. Many athletes self-reported symptoms and balance errors, even during asymptomatic periods before experiencing concussion, indicating unresolved issues. In the injury history survey, the baseline evaluations and injury histories of the participants classified into the SRC and non-SRC groups were compared. In the mBESS single-leg stance test (non-dominant foot), 84.21% (32/38) of the SRC group participants made errors, with an average score of 2.13 ± 1.52, whereas 55.55% (15/27) of the non-SRC group participants made errors, with an average score of 1.15 ± 1.35, showing a significant difference (*p* = 0.007). Additionally, significant differences were observed in the average number of ankle sprains (*p* = 0.027) and fractures (*p* = 0.048) between patients with and without a history of SRC. These findings indicate that athletes may have underlying issues even during normal periods before concussion. Moreover, the results highlighted the impact of previous concussions on motor control and injury risk. This underscores the importance of preseason baseline measurements using the SCAT to identify at-risk athletes and implement preventive measures. These findings align with the recommendations of the 6th International Conference on Concussion in Sport and suggest further refinement of concussion assessment tools.

## 1. Introduction

Sports-related concussions (SRCs) are reversible impairments in brain function caused by head trauma. Approximately 80–90% of patients with concussions recover naturally within 7–10 days without treatment [1]. However, symptoms can be diverse and prolonged, including cognitive impairment, sleep disturbances, vestibular dysfunction, and psychiatric symptoms, leading to a condition known as post-concussion syndrome [2]. In contact sports, SRC can recur upon returning to play, raising concerns about long-term consequences, such as chronic traumatic encephalopathy [3,4]. Therefore, the entire process, from the initial response to the SRC to the return to play, must be evaluated. Additionally, it has been reported that the risk of acute lower-limb injuries increases by 1.97 times within one year after sustaining a concussion, even if the symptoms have completely disappeared and clinical recovery has been achieved [5]. Patients with SRC may experience changes in motor control mechanisms, leading to an increased risk of secondary injuries and recurrent SRC, even in the absence of conscious symptoms. Therefore, establishing tailor-made baseline data for each athlete during the off-season is essential as a preventive measure. Understanding the preseason baseline conditions for each sport allows for objective medical interventions that contribute to the prevention of recurrent SRC and secondary injuries.

The current international consensus on managing sports concussions includes the use of the Sports Concussion Assessment Tool (SCAT). Various discussions on SRC have taken place, and the latest common understanding, agreed upon at the 6th International Concussion Conference in Amsterdam in 2023 [6,7], forms the basis for the revised evaluation tool known as the Sports Concussion Assessment Tool 6 (SCAT6, Child SCAT6). SCAT6 recommends a baseline assessment. Other useful tools for assessing suspected concussions, such as the Post-Concussion Symptom Scale and the Graded Symptom Checklist, also recommend calculating baseline scores under normal conditions and repeatedly evaluating them every few days to assess changes, similar to the SCAT [8]. In 2010, the National Collegiate Athletic Association adopted its Concussion Policy and Legislation, which is applied to more than 450,000 athletes annually. All athletes are advised to participate in a brain injury/concussion history survey, symptom evaluation, cognitive assessment, and balance evaluation before participating in collegiate sports [9]. However, these recommendations remain, and further research is needed to establish the necessity for baseline measurements. The SCAT is used when concussion is suspected, and if any applicable symptoms are present, concussion is generally suspected. The determination of SRC using the SCAT is based on the basic premise that an athlete is initially symptom-free. However, diagnosing SRC can be challenging if the athlete has a history of migraines, other medical conditions, or psychiatric disorders. Therefore, establishing a baseline to confirm that athletes do not exhibit any symptoms under normal conditions enhances the reliability of a post-concussion evaluation. Additionally, while many studies have identified sports in which SRCs occur frequently, considering the high risk of recurrent SRC and secondary injuries despite clinical recovery from SRC, it is essential that clinicians comprehensively understand the baseline pathophysiology during normal times. Therefore, measuring baseline data during non-injury periods before using the SCAT and the Ruler Drop Test or head trauma is presumed to be a more effective approach to SRC assessments in contact and collision sports.

This study focused on the importance of tailor-made baseline measurements when evaluating events after SRC and discusses the SCAT and the Ruler Drop Test. It aimed to conduct an off-season tailor-made baseline assessment using the SCAT to determine the usual condition of athletes, compare the baseline physical condition between those with and without a history of SRC, and examine the efficacy of the SCAT tool from a different perspective.

## 2. Materials and Methods

This study enrolled male rugby players from a university first-division league to undergo off-season tailor-made baseline assessments using the SCAT and investigate their medical history. Designed as a retrospective cohort study, it conducted baseline measurements and medical history investigations during the off-season. Its primary goal was to understand the athletes’ baseline conditions prior to concussion using the SCAT and evaluate the association between the risk factors of concussion history, baseline measurement results, and injury history. Furthermore, it aimed to assess the risk of concussion recurrence and the risk of secondary injuries. The assessments were performed in April 2023 during the off-season by a team consisting of a neurosurgeon, a physical therapist, and seven student trainers who had received standardized training. The student trainers received thorough explanations, specifically on the use of concussion assessment tools (SCAT) and the Ruler Drop Test and were trained in the required fundamental techniques. Athletes sidelined because of SRC, sports-related injuries, or trauma were excluded. Self-reported symptoms based on SCAT scores and the modified Balance Error Scoring System (mBESS) used in this study were evaluated off-field. Athletes were provided with private rooms to ensure a distraction-free environment, adhering to the measurement criteria of conducting assessments in such settings, where individual evaluations were performed. The measurements included the identification of the dominant foot, self-reported symptoms on the SCAT, the mBESS, and a Ruler Drop Test. Questionnaires were administered to collect historical information.

For self-reported symptoms, each player checked individual items from the 22 questions on the SCAT5 and rated their usual symptoms. To ensure judgment and assess post-SRC accuracy, the participants’ health was verified immediately before baseline measurement, and those in poor health on the same day were re-evaluated at a later date. The assessment aimed to understand what symptoms the athletes typically experienced according to the normal use of the SCAT5 and was conducted across seven levels: 0, none; 1–2, mild; 3–4, moderate; and 5–6, severe.

The mBESS followed the SCAT5 protocol using three stances: standing with both feet on a firm, horizontal surface; standing on the non-dominant foot; and a tandem stance with the non-dominant foot in the back, all performed with eyes closed. The experiments were conducted indoors with the participants standing barefoot on a firm surface. Each stance was held for 20 s, during which the number of errors was counted and scored. Errors were defined as moving hands off the hips, opening eyes, stepping, stumbling, falling, hip abduction exceeding 30°, and lifting toes or heels; any occurrence of these was considered an error. 

**Figure 1:**
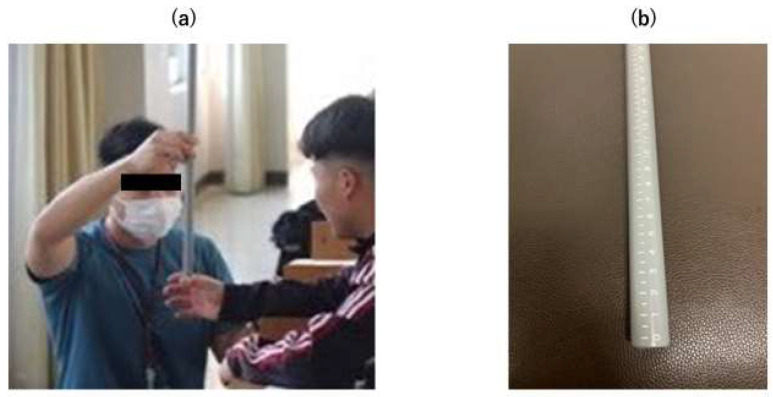
Ruler drop test: (**a**) The starting position was set at the point where the subject lightly opened the fingers of his dominant hand and placed the bottom edge of the reaction test stick (scale 0 cm) between the thumb and index finger. (**b**) The dropped stick was approximately 55 cm long, 2 cm in diameter, weighed approximately 115 g, and was made of plastic with a scale marked in 1 cm increments.

In this study, the Ruler Drop Test was performed to assess neural reflex function. The Ruler Drop Test is a straightforward and cost-effective clinical measure of reaction time (RT). In this test, the participants were required to catch a dropped measuring stick, and the distance it fell before being grasped was measured to provide a simple clinical RT. The clinical utility of the Ruler Drop Test was confirmed in previous studies, establishing that this RT test can be a component of a multifaceted concussion assessment battery [10,11,12,13]. The dropped stick was approximately 55 cm long and 2 cm in diameter, weighed approximately 115 g, and was made of plastic with a scale marked in 1 cm increments. The test was conducted thrice with the dominant hand using a dropped stick. The dominant hand was defined as the hand holding chopsticks or a knife. The test was conducted with the participant sitting in a chair with the elbow of the dominant hand resting at the corner of the desk for stabilization. The starting position was set such that the participant slightly opened the fingers of the dominant hand, and the bottom edge of the reaction test stick (marked at 0 cm) was placed between the thumb and index finger to ensure that the stick was dropped without any rhythm. The participants were instructed to quickly grip the reaction stick as it began to fall. The distance that the stick fell was measured in centimeters (Figure 1). After conducting one practice trial, the average distance from the three post-practice trials was used for the analysis. This distance was converted into RT in milliseconds using a specific formula [14].

$$RT\left(ms\right)=1000\times \sqrt{\frac{2\times distance(cm)}{980cm/S^{2}}}$$

In this study, the Mann–Whitney U test was used to evaluate the differences in various assessment items between athletes with a history of concussion (SRC group) and those without (non-SRC group). Data were collected from both groups for each assessment item (symptom score, mBESS single-leg stance, mBESS tandem stance, digit span test, Ruler Drop Test, and injury history). Given the potential non-normal distribution of the data for each assessment item between the SRC and non-SRC groups, the non-parametric Mann–Whitney U test was used. The significance level was set at *p* < 0.05.

This study was conducted with the consent of the participants in accordance with the Declaration of Helsinki and approved by the Ethics Committee of Fukuoka University (22-06-02, 15 July 2022). This study was supported by Fukuoka University (grant no. 23-03-M1).

## 3. Results

In 2023, baseline measurements and injury history surveys were conducted from 65 male rugby players from a university first-division league (average age, 19 ± 1.2 years; average playing experience, 9.9 ± 3.6 years). The analysis included average values and standard deviations for self-reported symptoms, mBESS scores, Ruler Drop Test distance and RT, and number of injuries for each type of trauma reported. Additionally, a comparative analysis was conducted between the SRC and non-SRC groups based on the baseline measurements and injury history data extracted from the surveys.

### 3.1. Sideline SRC Evaluation at Baseline and History of Injury Survey

For self-reported symptoms, 35.38% (23/65) of the participants reported symptoms, with an average of 1.5 ± 2.8 symptoms per player and an average symptom score of 2.66 ± 5.93. For the mBESS, no errors were observed in the tandem stance test, 72.31% (47/65) of the participants made errors in the single-leg stance test (non-dominant foot) with an average of 1.7 ± 1.5 errors, and 40% (26/65) made errors in the tandem stance test with an average of 0.82 ± 1.25 errors. The average distance for the Ruler Drop Test was 23.89 ± 6.85 cm, and the average RT was 218.52 ± 31.48 ms. Regarding the past medical history, the prevalence of prior SRCs was 58.46%; for ankle sprains, it was 76.92% (50/65); for fractures, it was 18.46% (12/65); and for knee injuries, it was 36.92% (24/65).

### 3.2. Comparison between Study Groups

**Figure 2:**
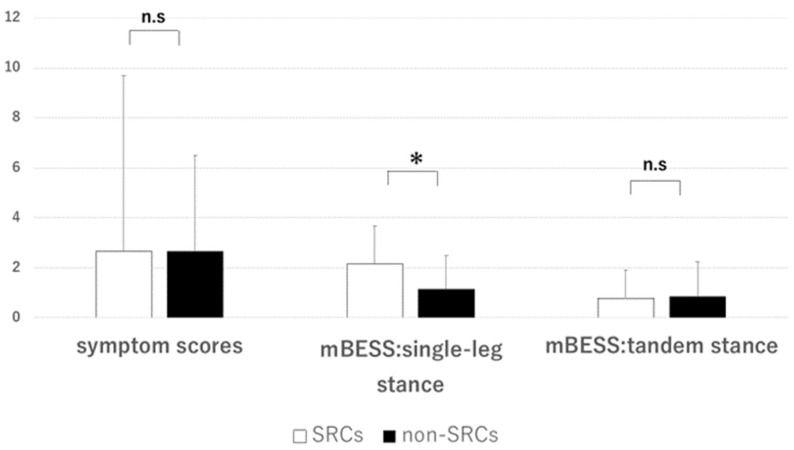
Comparison of baseline scores for the sideline concussion evaluation test between the SRCs group and the non-SRCs group for each assessment item (symptom scores, mBESS: single-leg stance, mBESS: tandem stance, digit span test). *: significant difference. n.s.: no significant difference. SRCs group: 38 players, average age: 19 ± 1.2 years, average years of play: 10.31 ± 3.87; non-SRCs group: 27 players, average age: 19 ± 1.2 years, average years of play: 9.26 ± 3.01. A significant difference was observed only in the mBESS for single-leg stance test.

**Figure 3:**
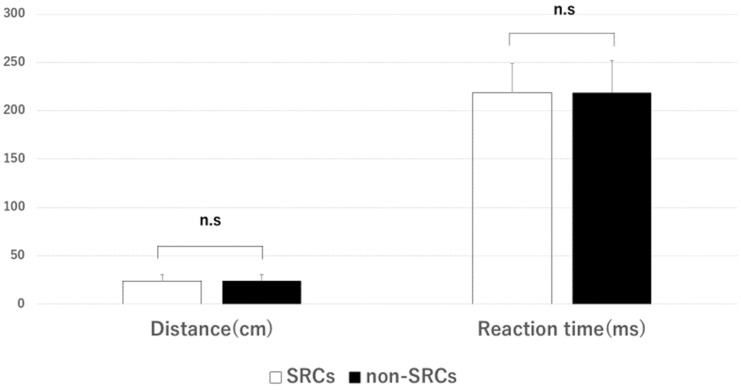
Baseline scores (distance and reaction time) for the Ruler Drop Test for both the SRCs group and the non-SRCs group. n.s.: no significant difference. SRCs group: 38 players, average age: 19 ± 1.2 years, average years of play: 10.31 ± 3.87; non-SRCs group: 27 players, average age: 19 ± 1.2 years, average years of play: 9.26 ± 3.01. No significant difference was observed between the two groups.

Among the SRC group (*n* = 38; average age, 19 ± 1.2 years; average playing experience, 10.31 ± 3.87 years), 28.94% (12/38) reported symptoms, with an average of 1.38 ± 3.08 symptoms and an average symptom score of 2.64 ± 6.86. In the non-SRC group (*n* = 27; average age, 19 ± 1.2 years; average playing experience, 9.26 ± 3.01), 44.44% (12/27) re-ported symptoms, with an average of 1.63 ± 2.28 symptoms and an average symptom score of 2.67 ± 3.85. No significant intergroup differences were observed in the number of symptoms (*p* = 0.233) or symptom scores (*p* = 0.188). In the mBESS single-leg stance (non-dominant foot), 84.21% (32/38) of the participants in the SRC group made errors, with an average score of 2.13 ± 1.52, whereas 55.55% (15/27) of the participants in the non-SRC group made errors, with an average score of 1.15 ± 1.35, demonstrating a significant difference (*p* = 0.007). No significant differences were observed in the tandem stance test results (*p* = 0.833). In the Ruler Drop Test, the SRC group had an average distance of 23.86 ± 6.69 cm and an average RT of 218.66 ± 29.86 ms, while the corresponding values in the non-SRC group were 23.91 ± 7.06 cm and 218.34 ± 33.62 ms, respectively. No significant differences were observed (*p* = 0.790; Figure 2 and Figure 3).

**Figure 4:**
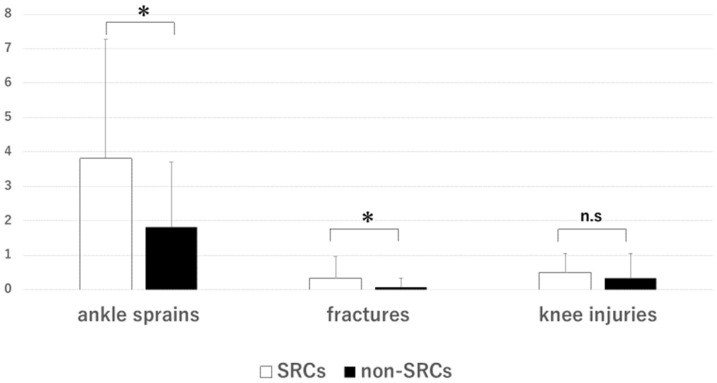
Number of injuries in the SRCs group and the non-SRCs group, categorized by medical history (ankle sprain, fracture, knee injuries). *: significant difference. n.s.: no significant difference. SRCs group: 38 players, average age: 19 ± 1.2 years, average years of play: 10.31 ± 3.87; non-SRCs group: 27 players, average age: 19 ± 1.2 years, average years of play: 9.26 ± 3.01. A significant difference was observed between the two groups in terms of ankle sprains and fractures.

The survey of injury history showed that the participants in the SRC group had an average of 3.79 ± 3.41 ankle sprains, 0.34 ± 0.61 fractures, and 0.49 ± 0.55 knee injuries. The non-SRC group reported an average of 1.81 ± 1.89 ankle sprains, 0.07 ± 0.26 fractures, and 0.33 ± 0.72 knee injuries. Significant differences in the average number of ankle sprains (*p* = 0.027) and fractures (*p* = 0.048) but not in the average number of knee injuries (*p* = 0.074) were observed between the groups (Figure 4).

## 4. Discussion

In this study of 65 university rugby players from a first-division league, 35.38% had self-reported symptoms, and 29.23% made errors in the digit span test, 72.31% in the mBESS single-leg stance test, and 40% in the mBESS tandem test. Our off-season tailor-made baseline measurements identified symptomatic and error-prone individuals for all measurement items except for the mBESS tandem test. The SCAT5 symptom evaluation lists 22 symptoms, ranging from specific symptoms such as “headache” to more abstract ones indicating the presence of chronic symptomatic individuals even in normal conditions. The top three most reported symptoms among the 35.38% with self-reported symptoms were “feeling drowsy”, “feeling fatigued or having low energy”, and “having headaches”. Migraine, a neurological disorder with high prevalence rates worldwide, has an incidence rate of 9.1–18.2% [15,16] and a lifetime prevalence of 28.6% [17] associated with “reduced quality of sleep” and “depression”.

Given the high rate (72.31%) of errors in the mBESS single-leg stance test, which evaluates postural control using vestibular and somatosensory inputs under closed-eye conditions, athletes with a history of SRC may have chronic vestibular system impairments. In SRC, impacts on the head and neck region are believed to result in a decreased function of sensory organs, such as vision [18], vestibular sensation, and proprioception. Impairments in the vestibular system are responsible for integrating sensory inputs and abnormalities in the sensory input [19]. The vestibular system integrates sensory inputs from the vestibular organs, neck, cerebral cortex, cerebellum, and eye movements, thereby influencing movement prediction, gaze stabilization, and postural control. Therefore, athletes with a history of SRC or those who are unaware of SRCs may have chronic vestibular system impairments. Despite clinical recovery, certain athletes with a history of SRCs have a higher risk of acute lower-limb injuries. Investigations among college athletes revealed a 2.48 times higher risk of acute lower-limb injuries within 90 days of return [20] and a 3.39 times higher risk of lower-limb injuries, leading to competition time loss within 90 days of return [21]. In this study, our classification of athletes into groups based on SRC history and the comparison of measurement item scores and injury history showed significant differences in the mBESS single-leg stance test and history of ankle sprains and lower-limb fractures, indicating a correlation between SRC history, mBESS scores, and injury history.

Previous studies reported reduced walking speed, decreased knee-joint range of motion, and altered power generation in the hip and ankle during running [22,23], resulting in changes in motor control functions after SRCs, as indicated by our study. In addition, post-SRC changes in motor control function can lead to decreased balance scores and increased injury risk [24]. Thus, even if neurological, neurocognitive, and balance assessments are normal and no symptoms are felt at rest or during a gradual increase in physical activity, great caution should be exercised when allowing athletes to participate in sports. 

The Ruler Drop Test is used to evaluate RT. In an intergroup comparison, the SRC group had a mean distance of 23.86 ± 6.69 cm, whereas the non-SRC group had a mean distance of 23.91 ± 7.06 cm. Although no significant difference was observed, the evaluation of cognitive function after concussion is recommended, and RT is considered an effective parameter [25]. The evaluation of post-concussion cognitive function is commonly conducted using computerized neurocognitive tests. However, simpler alternative evaluation methods are required because of the time required to collect baseline measurements. We consider the Ruler Drop Test an effective evaluation tool for baseline SRC measurements because it is simple and allows for the evaluation of more individuals in a short period.

In 2023, at the 6th International Conference on Concussion in Sports held in Amsterdam, the SCAT6 (including the Child SCAT6) was introduced as an update to the previous SCAT5. Six main changes were made to the content. In particular, the coordination and balance test contents showed the most significant changes. The recommended measurement sequence starts with a static balance test (e.g., mBESS or BESS) and progresses to single- and dual-task tandem gait measurements. Dual-task tandem gait adds complexity by including a cognitive component, such as counting backwards in 7 s while walking. Shigemori et al. [26] conducted a survey among 190 university athletes and found that 1.1% of the participants reported symptoms lasting over 15 min following a head injury but stated that they had not experienced a concussion (nor sought medical treatment), highlighting the presence of “unaware SRCs”. This suggests that individuals may not recognize an injury following a head impact, leading to a state of SRC where the individual believes that their symptoms have resolved but may still be in an “unaware SRC” state. 

The National Collegiate Athletic Association mandates baseline balance assessments for athletes at risk for SRC. We propose employing preventive measures for severe accidents in university athletes showing changes in motor control mechanisms post-SRC by conducting off-season tailor-made baseline measurements using the SCAT and Ruler Drop Test for athletes participating in amateur contact and collision sports.

## 5. Conclusions

In this study, tailor-made baseline measurements using the SCAT5 and the Ruler Drop Test were used to assess university athletes before the SRC off-season. Based on these results, the mBESS may be unreliable because of the different measurements conducted by the assessors and the potential influence of factors other than SRCs on performance. The results also revealed that athletes with a history of SRC or who were unaware of SRCs were at a higher risk of acute lower-extremity injury. Although the SCAT6 incorporates the evaluation of dynamic postural control through tandem gait, alternative methods for assessing static postural control are needed. The assessment of amateur contact and collision sports athletes, at least in off-season tailor-made baseline assessments, may contribute to the prevention of serious accidents.

## Data Availability

Data are contained within the article.
